# Testicular ultrasound: an emergency medicine perspective

**DOI:** 10.1007/s11739-025-03864-z

**Published:** 2025-03-18

**Authors:** José Mariz, Joaquin Martinez, Sheila Arroja, Michael Blaivas

**Affiliations:** 1 Emergency Department of ULS Braga, Braga, Portugal; 2 Medical School of University of Minho, Braga, Portugal; 3 European Federation Internal Medicine’s Ultrasound Working Group, Machelen, Belgium; 4 POCUSX, Braga, Portugal; 5 Venezuelan Society of Ultrasound in Medicine (AVUM), Caracas, Venezuela; 6 Latin American Study Group on Multisystemic Ultrasonography in Pediatrics (USPed Latin America), Buenos Aires, Argentina; 7 University of South Carolina School of Medicine, Columbia, SC USA

**Keywords:** Acute scrotum, Torsion, Testis, Doppler

## Abstract

Ultrasound of the scrotum plays a crucial role in assessing acute scrotal conditions in the Emergency Department. Although the Emergency Physician and Intensivist have shared responsibility for the care of the critically ill patient, the Emergency Physician typically uses Point-of-care Ultrasound in a broader range of applications than the intensivist to include advanced abdominal, obstetric, testicular, musculoskeletal, and ocular ultrasonography. Acute scrotum refers to the sudden onset of scrotal erythema, swelling, or pain, and it is not a rare condition in the Emergency Department. Prompt intervention is required in cases of testicular torsion or rupture, and ultrasound of the scrotum has high utility for emergency physicians seeing acute scrotal complaints with any frequency. However, the incidence of acute scrotum incidence is low compared to other disease states requiring ultrasound diagnosis. This presents a problem when considering ultrasound training of Emergency Physicians for ultrasound of the scrotum in a Point-of-care perspective. With this narrative review, we will attempt to raise the awareness of emergency medicine doctors to the importance of ultrasound of the scrotum in the Emergency Department. We will also discuss educational aspects in testicular ultrasound and the use of contrast-enhanced ultrasound. Finally, we propose an algorithm for action.

## Introduction

Ultrasound (US) use by Emergency Physicians (EPs) is expanding both in academic and private practice. Applications, such as trauma, abdominal, endovaginal, and cardiac US, are flourishing due to urgency in diagnosing a range of organ and life-threatening disease states. Although the EP and intensivist have shared responsibility for the care of the critically ill patient, the EP typically uses Point-of-care Ultrasound (POCUS) in a broader range of applications than the intensivist to include advanced abdominal, obstetric, testicular, musculoskeletal, and ocular ultrasonography [1, 2].

Using POCUS, the EP performs all image acquisition and interpretation at the point of care and uses the information immediately to address specific hypotheses and to guide ongoing therapy and intervention. This requires that the EP has skill at image acquisition, image interpretation, and the cognitive elements required for results integration into clinical decision-making. However, unlike the standard workflow of traditional consultative ultrasonography, the emergency department (ED) POCUS examination is often focused in scope and goal directed; or, depending on the clinical situation, available time, and skill of the operator, it may be as comprehensive as the standard consultative examination [3] .

In the ED, US of the scrotum plays a crucial role in assessing acute scrotal conditions. While traditional consultative ultrasonography typically involves a thorough evaluation of the entire male genital system, the focus of US performed by an EP in acute cases is more targeted. The EP uses US to rapidly narrow the differential diagnosis, allowing for prompt decision-making—either to pursue conservative management or expedite surgical intervention [4].

Acute scrotum refers to the sudden onset of scrotal erythema, swelling, or pain. It is not a rare condition in the ED [5]. The acute scrotum, particularly, in children and younger men is a challenging condition for EP, often hijacked by the term ‘potential testicular torsion’ in recognition of the urgency to treat and not miss this time-critical condition [6].

Since 1999, when one of the authors (MB) published the first cases for emergency screening US examinations of testicular torsion [7], the use of US by EP and internists dedicated to emergency medicine (EM) has increased exponentially. However, US of the scrotum, particularly the acute scrotum, remains an unexplored territory in EM. Studies on EP use of testicular ultrasonography have been limited to several case reports and retrospective studies that have compared its accuracy with surgical findings, nuclear medicine studies, and radiology US examinations, and that did not change much, since the first reviews on the subject were published [3, 4, 8].

With this narrative review, based on the extensive experience of the authors from different geographic medical practices, we will attempt to present the current state of the art of the main applications of scrotal US from the perspective of EM. At the end, we propose an algorithm for action.

### Technology

Ultrasonography of the scrotum includes greyscale, color, and pulsed-wave Doppler evaluation of testicles, epididymides, and the scrotum [9]. Optimal scanning is performed using a linear high-frequency (> 7 MHz) transducer with broad bandwidth (6–12 MHz or higher). The equipment should be capable of performing color, power, and pulsed Doppler scans [4, 10].

The US exam of the scrotum in the ED causes some challenges, which are mainly related to the intimate nature of the examination and potential for serious outcome, and which often cause great anxiety [4].

**Fig. 1:**
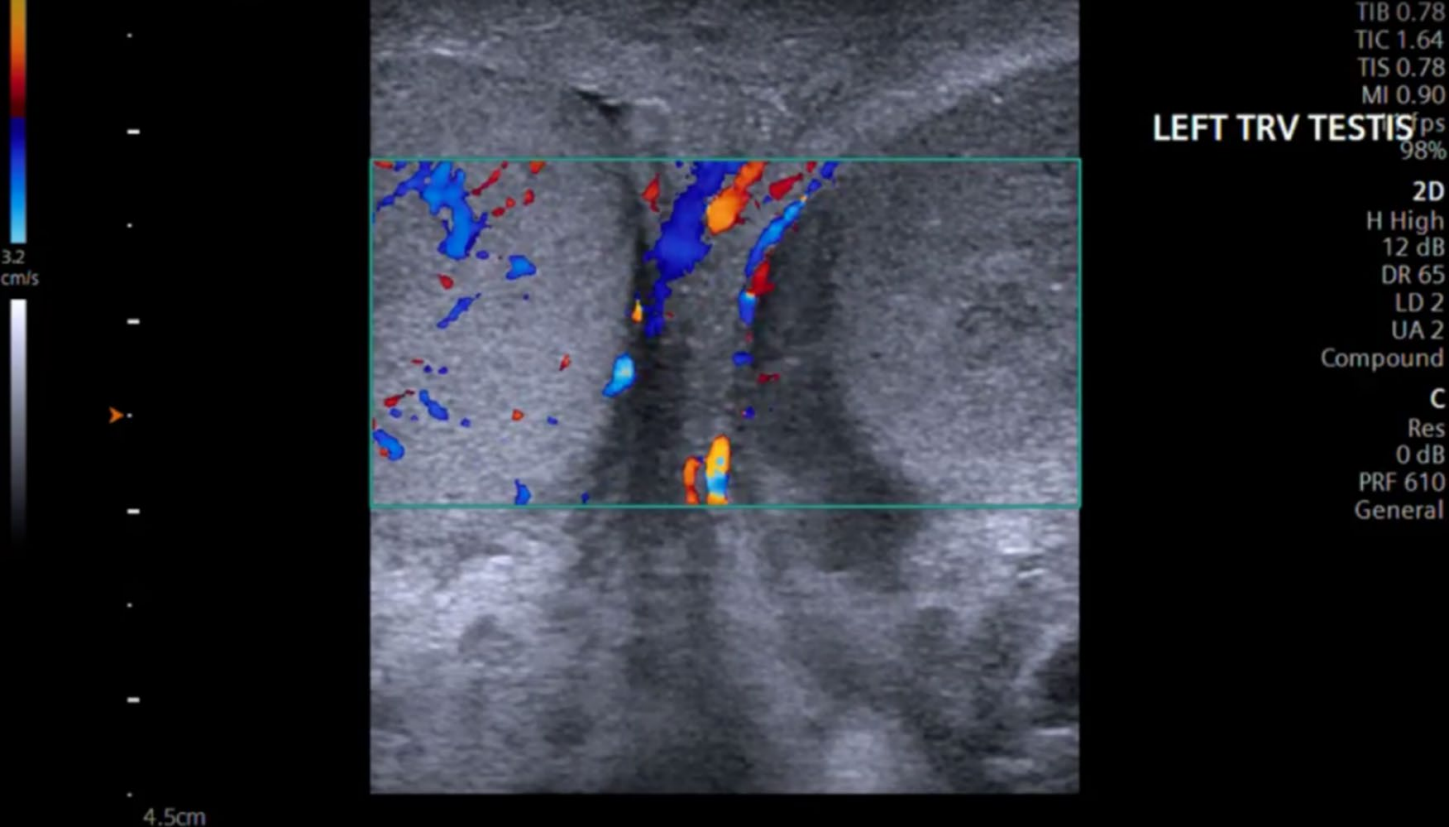
A midline transverse image, including a portion of each testis, is essential in the comparison of echotexture and vascular flow. Here, the right testicle shows asymmetric flow in relation to the left testicle, using Color Doppler with a velocity scale in the order of 3.2 cm/s

Therefore, a special care must be taken to place the patient in a comfortable position to ensure optimal positioning for correct ultrasonographic evaluation. The patient should be placed in a supine position, in which the hips and knees are flexed and externally rotated (frog-leg position), so that the penis is against the abdomen, to keep it out of the examination field and the scrotal sac is properly supported by a towel from beneath. The testes are imaged in the sagittal and transverse planes. A midline transverse image, including a portion of each testis, is essential in the comparison of echotexture and vascular flow (Fig. 1) [11]. Both testes should be scanned in the sagittal and transverse planes from one edge of the testes to the other.

Attention must be paid to correct use the color and power Doppler modes. Color Doppler is a color-based display of blood flow in vessels of the scrotal contents. It assigns one color to flow toward the transducer and another to flow away from the transducer. The ‘‘color map’’—the scale that dictates what colors are seen—can be changed and this mode is sensitive to the direction of blood flow. The notion of the magnitude of the scale for Doppler assessment of blood flow in the testicle is very important, since the expected flow velocities are in the order of 3–5 cm/sec in the normal testes. Power Doppler is traditionally direction insensitive, making it more likely that blood flow is registered. The ability to measure the power of the Doppler signal rather than the Doppler frequency shift significantly enhances its sensitivity. The result is that power Doppler is the mode of choice over color Doppler to pick up slow-moving blood, such as seen in ovaries and testicles [12]. It is not uncommon to scan a patient’s testicle using color Doppler and initially be left wondering whether there is any flow in the organ. After changing to power Doppler, however, the amount of flow that is seen almost always increases in the normal testicle [3].

It is also important to be facile with adjustment of color Doppler parameters as many modern US machines are quite sensitive to low flow states if properly adjusted.

### Pathologic findings

#### Testicular torsion

Testicular torsion is a urological emergency that requires an accurate and timely diagnosis for prompt and adequate management. It occurs due to the rotation of the testicle around the spermatic cord, triggering vascular compromise that, if sustained, can cause permanent damage to the testicle due to ischemia and potentially loss of fertility [13]. The degree of damage will be determined by the duration of the event and the degree of rotation or torsion of the cord around its own axis, and it is worth highlighting the susceptibility of the testicle to ischemia due to its terminal flow, without anastomosis between the testicular arteries, and the inability to distend the tunica albuginea, which limits compensatory expansion [14].

There are two types of torsion: supravaginal or extravaginal, which occurs in the prenatal and neonatal period, and intravaginal, which is the most frequent and has a higher incidence in adolescence. They can be classified as total, when the rotation of the cord is equal to or greater than 360°, and incomplete, where there may be residual flow to the testicle below this degree of torsion [14, 15].

Testicular torsion can occur at any age; however, it is more common during childhood and adolescence, with an incidence of 4.5 per 100,000 men between 1 and 25 years of age [16]. However, testicular torsion has an incidence that is low compared to other disease states requiring US diagnosis. This presents a problem when considering US training of EPs for testicular torsion.

The clinical presentation typically involves pain of abrupt onset and edema in the hemiscrotum corresponding to the affected testicle (depending on time since onset), vomiting, nausea, negative Prehn sign, absence of the cremasteric reflex, and even radiation of the pain to the ipsilateral iliac fossa [13].

Although testicular torsion is a clinical diagnosis, scrotal Doppler ultrasonography is often used as a diagnostic adjunct [17]. Since the seminal work of Burk et al. in 1990, the use of the simple criterion of absence or presence of demonstrable intratesticular arteries, using Color Doppler, has reached values of accuracy of the order of 97% [18].

The importance of timely diagnosis for surgical resolution of the condition must be emphasized, since the delay in identifying the torsion inevitably results in the loss of the testicle. The salvage rate of the affected testicle is up to 100% in the first 6 hours, 75% at 12 hours, but less than 50% from 12 to 24 hours. The combination of B-mode and Doppler assessment through US in testicular torsion yields a sensitivity of 81 to 100%, and an accuracy of 90 to 98% [10, 19]. US is the method of choice given its availability and potential for rapid interpretation, as the case warrants. Additionally, scrotal POCUS performed by EP appears to be an accurate tool to detect testicular torsion both in children and adults with acute scrotum and saves time compared with radiology US [7, 19].

**Fig. 2:**
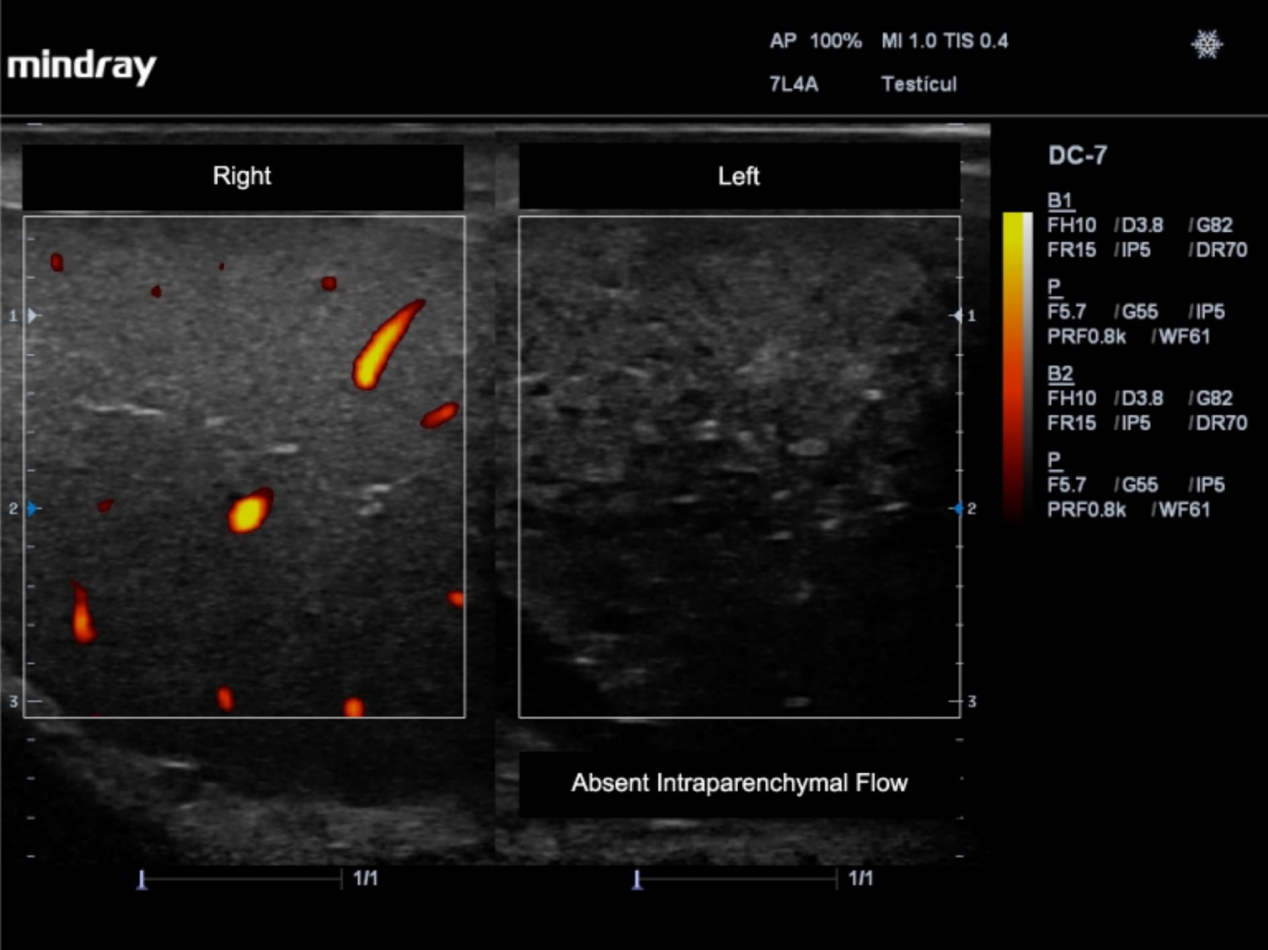
Absent flow and changes in echogenicity in the left testicle due to testicular torsion. Power Doppler was applied and a comparison with the right non-affected testicle is shown

Regarding the US study, there are important findings for the identification of the entity, emphasizing that all findings in the affected testicle must be compared with the contralateral one:

• Increase in volume and changes in echogenicity of the affected testicle and ischemia of the epididymis in relation to the same contralateral structures [15, 20].

• An abnormal testicular axis, often described as a "bell-clapper" testicle, refers to a condition in which the testicle is abnormally positioned within the scrotum, typically lying horizontally or displaying antiparallelism to its normal vertical axis. This is due to a congenital anomaly where the parietal plate of the tunica vaginalis fails to properly adhere to the testicle, and there is an incomplete connection between the epididymis and the testis [15, 20].

• Decreased or absent flow in the affected testicle (Fig. 2) [15, 20].

• "Whirlpool" sign, produced by the rotation of the spermatic cord at its torsion point, either in the inguinal canal or paratesticular [15, 20].

#### Testicular masses

Identification of testicular cancer typically should not be considered the goal of emergency ultrasonography. However, testicular masses still present to the ED and among the associated symptoms are scrotal pain, with or without volume increase, focal increase in the consistency of the testicle, and extratesticular signs such as gynecomastia, abdominal pain, inguinal pain and volume increase, or dyspnea [21]. In these cases, risk factors, such as alterations in testicular descent, family or personal history of previous testicular cancer, infertility, smoking, and exposure to chemicals, should be considered. The risk association between testicular microlithiasis and cancer is still controversial, despite its previous mention and consideration of its relationship with oncological pathology [22, 23].

Regarding epidemiology, testicular cancer is one of the most common tumor lesions in men between the ages of 15 and 40 years, although it represents only 1% of adult neoplasias and 5% of urological tumors. Of these, up to 95% are germ cell tumors [21, 24] .

**Fig. 3:**
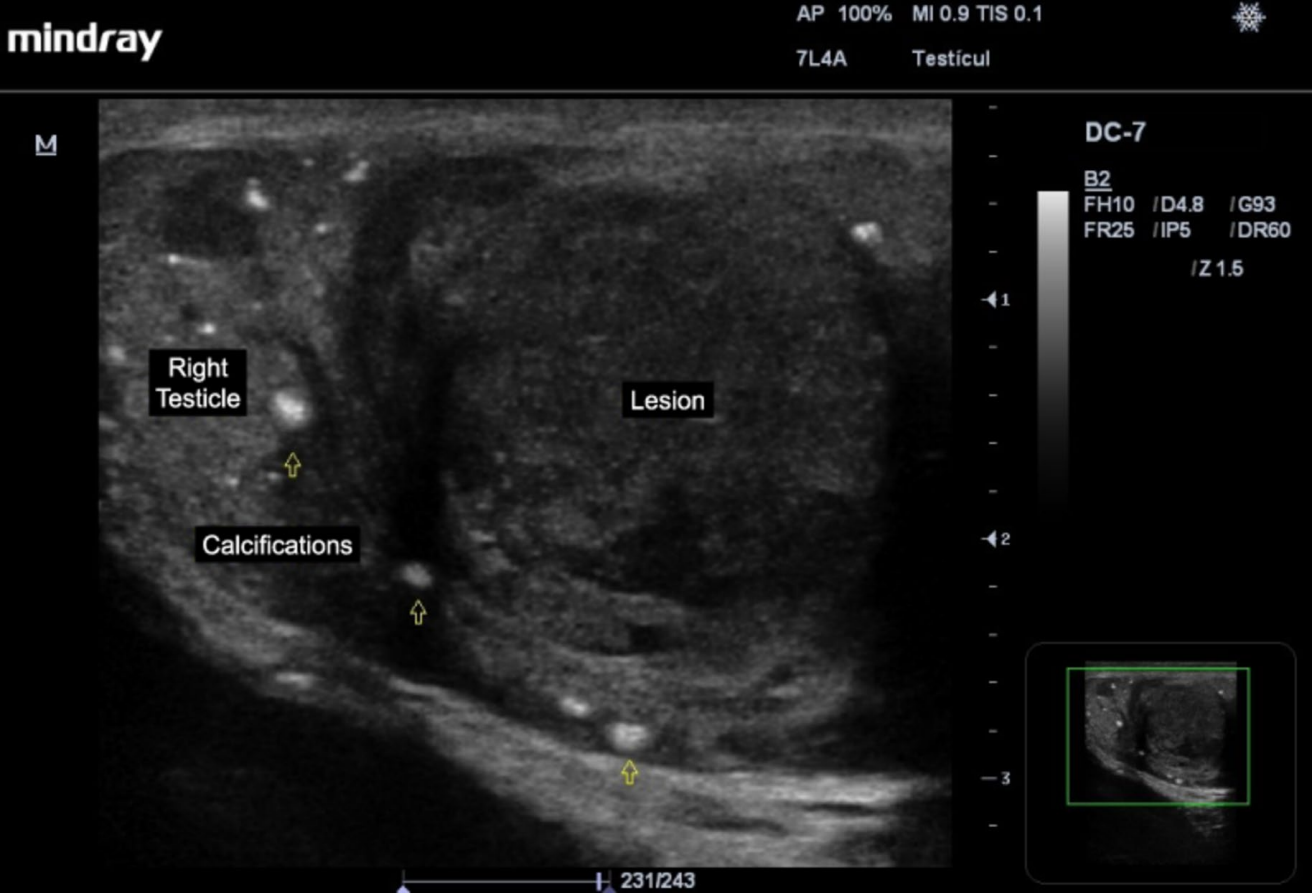
Seminoma is observed as a homogeneous hypoechoic lesion, with calcifications (open arrows) and intralesional anechoic areas corresponding to necrosis

Of the germ cell tumors, the most common are seminomas (up to 50%), which are observed as homogeneous hypoechoic lesions, with calcifications inside, which can be multilobulated, and with intralesional vascularization present on Color Doppler study, and can take up the entire length of the testicle, and also have intralesional anechoic areas corresponding to necrosis (Fig. 3) [3]. Non-seminomatous germ cell tumors have a more heterogeneous pattern and a greater tendency to intralesional necrosis [21, 25, 26].

US is an excellent tool for the diagnostic guidance of testicular masses, with a sensitivity of 92–98% and a specificity of 95–99% [22], which offers confidence regarding timely referral of the case for management. In turn, the clinical context associated with the examination must always be considered to focus on the relevant differential diagnoses, such as testicular abscesses, rete testis ectasias, focal orchitis, segmental testicular infarcts, among others [23], for which the Color Doppler assessment is of great help for early guidance.

#### Testicular trauma

Genitourinary injuries associated with trauma represent up to 10% of the cases in these type of patients [27]; and among them, scrotal trauma comprises 1% of patients with trauma. In such cases, US is the first-line imaging resource for identifying the injury, its location, and its extension, to decide on the appropriate and timely management to ensure the best management [28].

It is important to mention the type or mechanism of trauma that may be involved:

- Closed trauma: The most common (up to 80%), usually unilateral and more associated with sports activities, falls, traffic accidents, and straddle injuries.
- Penetrating trauma: There is identifiable loss of scrotal integrity, and it occurs in stab or gunshot wounds, bites, or self-mutilation.
- Scrap trauma: Rare, associated with high-energy impacts (heavy machinery, traffic accidents) [28].

For a better understanding of the effect of trauma on the structures involved, it is useful to divide them between extratesticular and intratesticular injuries. Among the extratesticular injuries are scrotal abrasions, and hydroceles or hematoceles, which can be present in up to 25% of cases of major trauma. Hydroceles are anechoic liquid collections, which separate the parietal and visceral layers of the tunica vaginalis, without septa or suspended elements. Their presence in major trauma should raise concern for rupture of the bulbar urethra, as urine may extravasate into the scrotum and imitate a hydrocele [29].

Hematoceles are seen as liquid collections between the parietal and visceral layers of the tunica vaginalis but are generally more heterogeneous in terms of their echogenicity. They have variable appearance which depends on their chronicity which impacts evolution and retraction of the clot. The appearance is hyper or isoechogenic in relation to the testicle in acute phases; subsequently, their echogenicity decreases with concomitant appearance of septa, without vascularity, in later stages [24, 29]. Large hematoceles can generate a decrease in testicular blood flow due to the extrinsic compression exerted and imitate testicular torsion. In turn, in hematoceles of this type, surgical exploration should be considered looking for lesions of the tunica albuginea [29].

The tunica albuginea is an important indicator in intratesticular injuries. The tunica albuginea is a fibrous layer that completely covers the testicle and gives it its contour [30], and by US, it is seen as an isoechogenic line around it forming its external testicular border. In intratesticular injuries, the identification and verification of the integrity of the tunica albuginea is the key to the decision of surgical management in testicular trauma, so its status should always be documented in these assessments [28], which will allow us to differentiate these injuries into two groups, such as testicular fractures and ruptures.

In testicular fractures, the tunica albuginea will be intact, without disruption of the same, maintaining the usual oval shape of the testicle, with a hypoechoic band that extends through the testicular parenchyma, without intralesional vascularization, and in most cases with an associated hematocele [29, 31]. The direct identification of the fracture line is not so frequent, being able to be identified in 17% of cases [32]. If there is adequate vascularization of the testicular parenchyma, conservative management is chosen, with US monitoring.

**Fig. 4:**
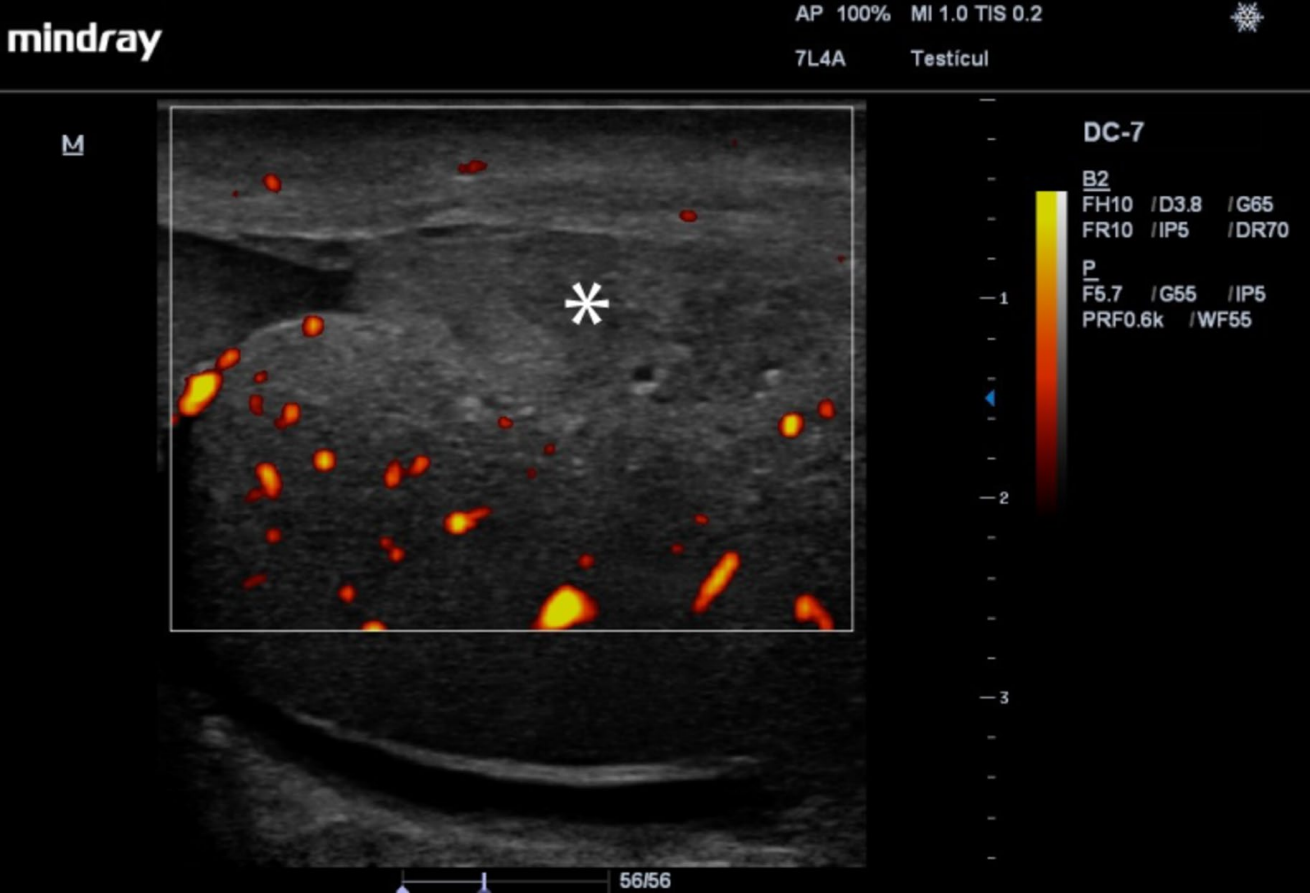
Testicular rupture with interruption of the tunica albuginea, extrusion of the testicular parenchyma (asterisk) through the point of disruption of the same, and a heterogeneous testicular parenchyma. The Power Doppler evaluation shows the absence of vascularization in the affected territory

With testicular ruptures, US may show interruption of the tunica albuginea, possibly with extrusion of the testicular parenchyma through the point of disruption of the same, which causes loss of the oval shape of the testicle, and a heterogeneous testicular parenchyma. In addition, during the Doppler-Color evaluation, injury may be evidenced by absence of vascularity in the affected territory, which is generated by the injury on the tunica albuginea and the relationship between it and the tunica vasculosa (Fig. 4) [28, 29, 31–33]. The combination of the elements described can give us a sensitivity close to 100% and a specificity of up to 93.5% for the diagnosis of testicular rupture [2], which is important for the surgical management and prognosis of the affected testicle, with success rates close to 90% with surgery in the first 72 hours, and only 45% after this period [27]. It is important to note that a small fracture may be difficult to detect as tunica albuginea disruption on US and secondary signs such as a change in blood flow locally may be the only evidence.

#### Orchiepididymitis

Orchiepididymitis is an inflammatory process involving the epididymis and testicle, most often of infectious etiology, and is the most frequent cause of testicular pain in adults, with more than 600,000 cases diagnosed per year in the USA. In up to 35% of cases, the etiologic agent is sexually transmitted organisms, such as *Chlamydia trachomatis* and *Neisseria gonorrhoeae*. Physical examination reveals an enlarged, painful testicle, with thickening and erythema of the skin of the hemiscrotum, fever, and in turn, thickening and pain on palpation of the spermatic cord [34].

Due to retrograde bacterial dissemination, the tail of the epididymis is the first affected site, spreading from there to the rest of the epididymis and the testicle [35]. In the B-mode assessment, we will observe, according to the extension of the infection, an increase in the size of the epididymis and the testicle with heterogeneity of the same due to edematous and hemorrhagic changes [36]. Associated findings can include paratesticular collections such as hydrocele or pyocele and thickening of the wall of the affected hemiscrotum. If the infection is left unchecked, the main complication is the formation of abscesses, which will be evident as hypoechogenic lesions in the epididymis or in the testicle [9].

**Fig. 5:**
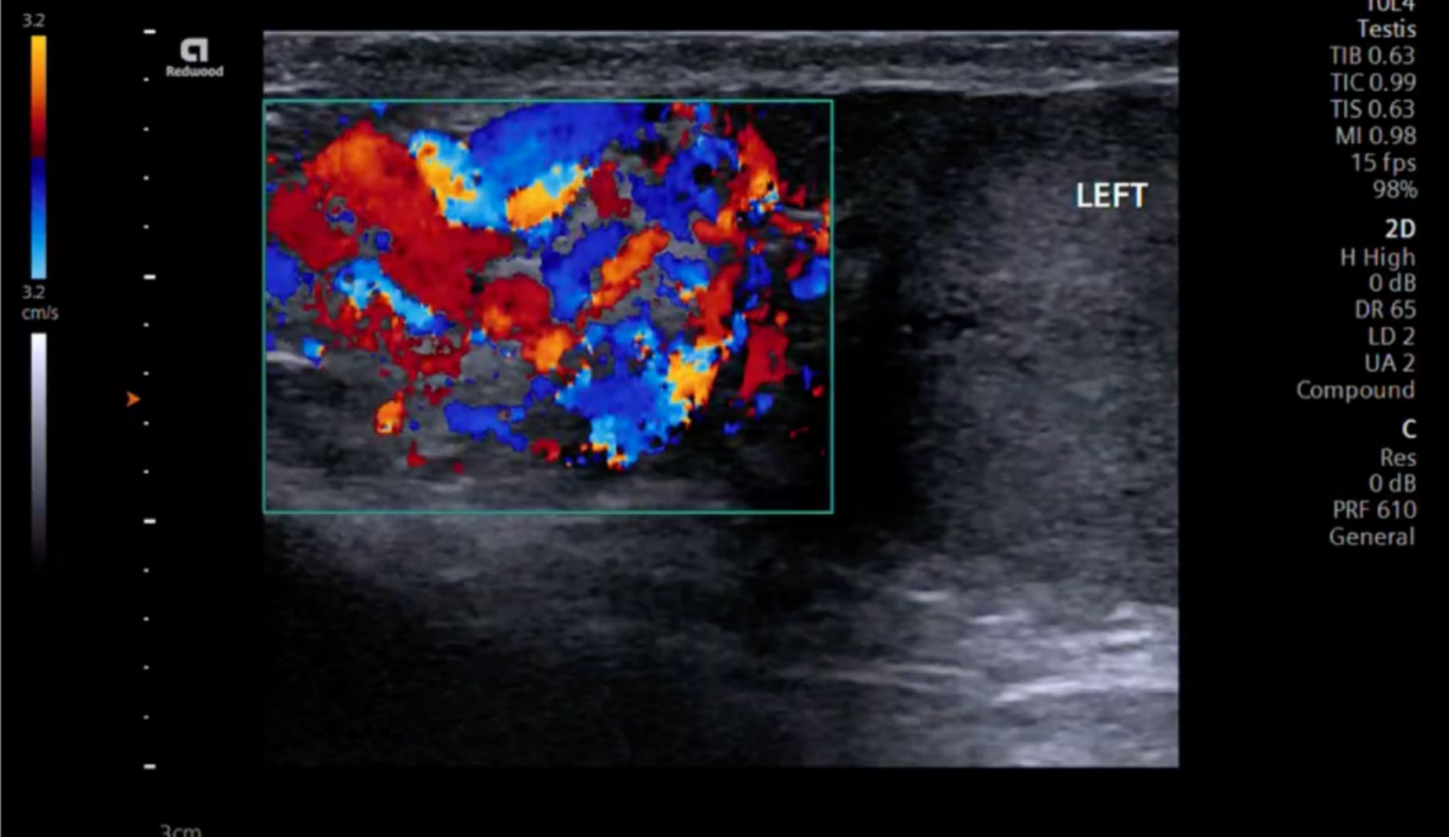
Epididymitis show an increase in the Color Doppler signal with respect to the head of the epididymis, due to the hyperemia associated with the infectious process

It should be noted that in the B-mode evaluation, both Orchiepididymitis and testicular torsion can be very similar. This is why a Color Doppler evaluation is necessary for the purpose of making a differential diagnosis. In these cases, US shows an increase in the intraparenchymal signal with respect to the contralateral testicle and epididymis, due to the hyperemia associated with the infectious process, with a sensitivity of up to 100%, which is of invaluable help in guiding us (Fig. 5) [4, 9, 36]. A comparative analysis of findings between the affected epididymis and testicle and their contralateral pairs should always be made. In rare cases, detorsion hyperemia can be mistaken for orchitis. However, the clinical scenario in such cases would include a change in degree of pain. Given the ease of POCUS scrotal scanning, a repeat scan can always be performed to rule out detorsion hyperemia, since it will typically last less than 15–30 minutes. In fact, one of the greatest benefits of POCUS over consultative radiology scans for evaluating the acute scrotum is the ability to rescan the patient even as quickly as 15–30 minutes after the initial scan to confirm findings, look for progression of vascular compromise, and confirm successful detorsion. This is exceedingly burdensome for radiology departments but may lead to incorrect diagnosis in some cases.

#### Torsion of testicular appendages

The testicular appendage has its embryological origin in the upper part of the paramesonephric duct and is located in the upper part of the testicle. Although they were previously considered congenital anomalies, recent studies report their presence in the majority of the male population [37], unilaterally in up to 92% and bilaterally in up to 69%. Its appearance on US is that of a paratesticular structure with echogenicity similar to that of the head of the epididymis, 1 to 7 millimeters in length, and most commonly oval and sessile, although in some cases pedunculated, and even calcified, to a lesser extent, and it is important to mention that there are also epididymal appendages [38].

Torsion of the testicular appendages represents the most frequent cause of acute prepubertal scrotum, comprising up to 60% of cases [39], with physical examination showing pain in the affected hemiscrotum, with increased consistency or palpable nodule in the upper testicular pole, and presence of a blue dot sign corresponding to the infarcted appendix, with preservation of the cremasteric reflex [40], the latter helping in the absence of US to make the differential diagnosis with testicular torsion itself, which will always be the headache, given the similar presentation of both entities in many cases.

The US examination will show differences between the acute and chronic phase. Initially, they will be observed with decreased echogenicity and increased dimensions, up to 1.7 millimeters in length, subsequently increasing their echogenicity (hyperechogenic in relation to the epididymis and ipsilateral testicle) and decreasing their dimensions, adopting an atrophic appearance in some cases. The Doppler-Color study will show the absence of intralesional blood flow, with loss of the vascular pedicle, and in some cases, increased vascularization in the underlying testicular plane due to hyperemia. Among the extra-appendicular findings, reactive hydrocele and focal thickening of the scrotal wall are the most common [39].

#### Scrotal infection

Fournier's gangrene is a form of necrotizing fasciitis that affects the perineal and abdominal region and constitutes a urological emergency due to its high mortality rate, which can reach up to 40% [41]. Originally described in 1883 by Dr. Jean Alfred Fournier, this infection usually has starting points at the genitourinary, anorectal, or cutaneous level; predisposing factors, such as diabetes mellitus, alcoholism, kidney failure, prolonged hospitalization, among others, are often present; and is polymicrobial in nature, with *Escherichia coli, Bacteroides, Streptococcus, Staphylococcus, Clostridium,* and *Klebsiella* being identified among the most frequent pathogens [42, 43].

At the clinical assessment, in the initial stages, the diagnosis can represent a challenge. What we mainly find is perineal edema, pain, erythema, and subcutaneous crepitation, and among the systemic manifestations are fever, tachycardia, and hypotension, with a high risk of progression to septic shock [44].

**Fig. 6:**
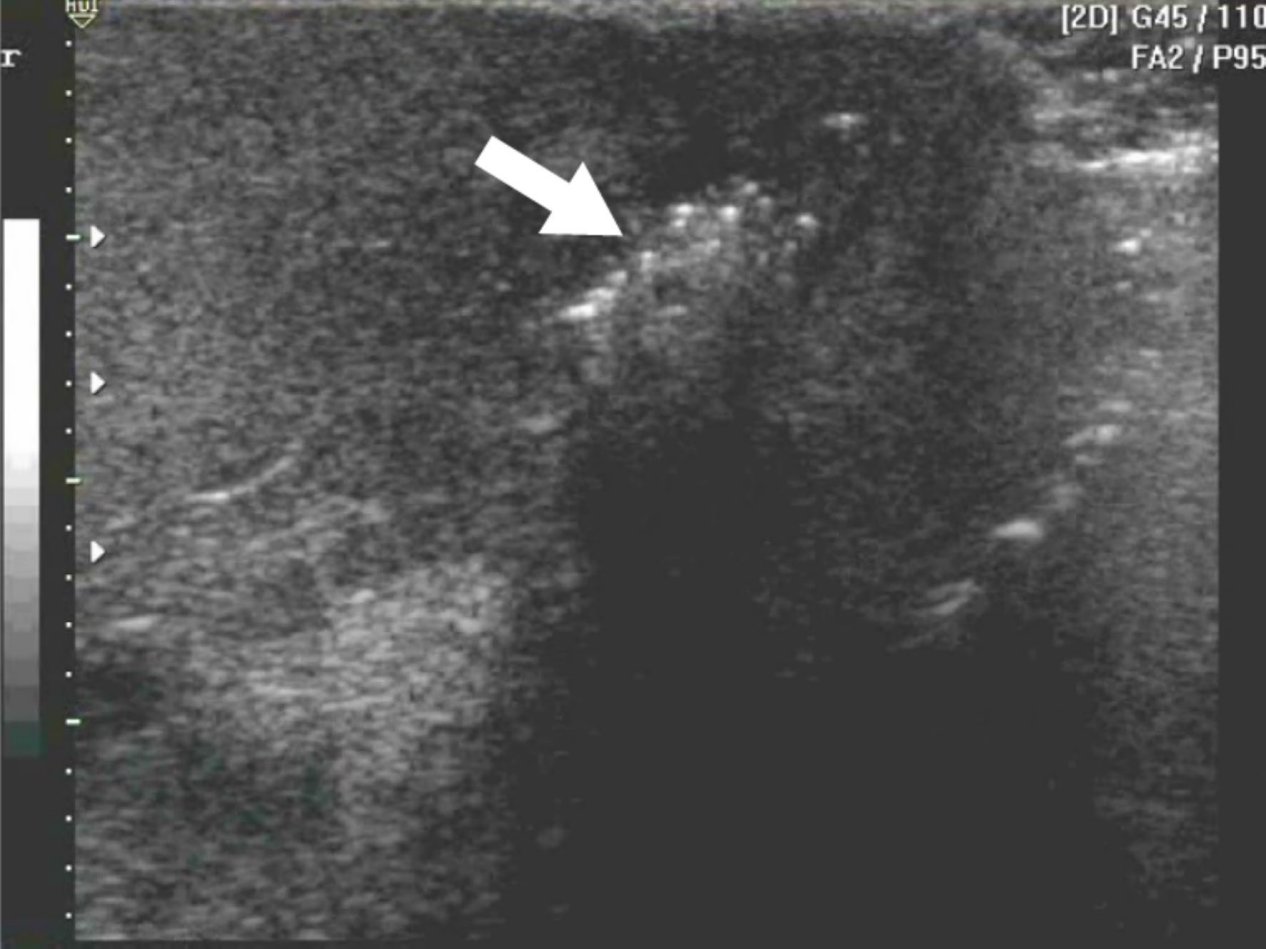
Fournier’s gangrene: multiple hyperechoic foci with a "dirty shadow" and subsequent reverberation artifacts associated with the gas formed by the pathogens involved (arrow), which constitutes the pathognomonic sign in this entity

The US evaluation may show several findings including thickening of the scrotal wall with multiple hyperechoic foci with a "dirty shadow" and subsequent reverberation artifacts associated with the gas formed by the pathogens involved, which constitutes the pathognomonic sign in this entity (Fig. 6) [45, 46], as well as the presence of reactive hydrocele, edema of the spermatic cord [42, 45], "cobblestone" sign at the subcutaneous level, due to the edema of fatty globules surrounded by liquid. And the "snow globe" effect, which is heterogeneous subcutaneous material with a swirling appearance [47]. US in necrotizing fasciitis, such as Fournier's gangrene, can provide a sensitivity of 88% and a specificity of 93% [48], which makes this imaging modality an important diagnostic resource in these cases.

### Educational aspects

The scrotum, like the thyroid, is an easily accessible and superficial structure. Unlike the thyroid, modesty considerations are required for scrotal scanning, especially in educational settings. Most pathology can be learned from video of cases from actual patients, such as hernias, infections, and trauma. However, actual practical experience is exceedingly helpful when torsion is being considered, especially when it is partial and has relatively vague findings. The pressure of evaluating possible torsion and the administrative and/or legal ramifications of missing torsion requires greater confidence than can be found from reviewing still images or video. An excellent model for torsion comes in the form of male volunteers who can manually compress their spermatic cord fully or partially cutting off flow to the testes. First used in a course for EM residents 25 years ago, it provides the clinicians with the actual experience of searching for and proving the existence of both venous and arterial blood flow in each testicle, coupled with a randomly inserted and blinded cord compression which leads to abnormal blood flow results. While this model misses the potential to identify a twisted spermatic cord to prove an anatomical cause for possible torsion, the reality is that little matters in the face of either certain torsion or its absence.

### Contrast-enhanced ultrasound (CEUS)

CEUS can be exceedingly useful when ruling out testicular torsion with inadequate color and or pulsed-wave Doppler sensitivity [28, 49–51]. This can occur with insensitive devices like some POCUS carts and also hand-held US machines. Additionally, such difficulty can be due to patient factors, such as morbid obesity, swelling, or prepubescent children. Regardless of the cause, if both testes seem to have little if any blood flow on color Doppler, US contrast may greatly enhance the clinician’s ability to evaluate presence of blood flow. Given that, in most cases, a control (contralateral) side is readily available for comparison, CEUS use is unlikely to create falsely normal or present blood flow when in reality some degree of torsion is present.

Regulations regarding CEUS use vary significantly from region to region with clinicians often left confused by the differences in allowed use. Additionally, the concept that contrast, which circulates throughout the entire body, can only be used for a specific organ or set of organs puzzles many emergency providers. In some locations, off label use, especially in case of organ threatening emergency is supported by law, in others, the barriers to use may be absolute. When using contrast, safety should be considered by the clinician and that requires some familiarity with the agent being used. Timing of contrast enhancement of blood flow should be known so as to not miss the optimal imaging window. While radiology traditionally does not image contrast without specific contrast settings on an US machine, this is not actually needed to benefit from CEUS. Even standard gray-scale imaging can be significantly enhanced by contrast administration and color Doppler use. Additionally, many newer POCUS devices come or have available US contrast settings allowing for optimized imaging.

## Conclusion

### Acute scrotal pain evaluation algorithm

One of the most attractive aspects of a POCUS-driven acute scrotum evaluation is that the clinician is not undertaking a general scrotal scan with a myriad of potential findings, but rather a clinical scenario driven search for testicular fracture, scrotal infection/inflammation, hernia, or torsion/blood flow compromise. It is important to keep in mind that unlike a traditional US examination of the scrotum, a POCUS examination does not happen in a vacuum. The clinician performing the scrotal US has already obtained a history from the patient and performed a physical examination. Thus, a narrowed differential is immediately targeted at the start of the examination. Signs of fluid or air are sought in the skin or scrotal contents and is usually suggested by warmth or redness of the scrotal skin on examination along with corroborating history. Herniation may be targeted on examination when suggested by history and physical and should focus mainly on evidence of bowel incarceration in the scrotum.

An evaluation with color/power and then pulsed-wave Doppler, especially when compared to the contralateral side, allows the clinician to identify or rule out epididymitis, orchitis, and testicular torsion. For testicular torsion, it is imperative to document several instances of both venous and arterial blood flow on pulsed-wave Doppler to avoid missing partial torsion, which can present with a dampened arterial wave from which appears venous—a testicularly fatal mistake if the operator makes the assumption that if the low-pressure venous system is functional, so surely is the arterial and no torsion is present. Testicular fracture may be evidenced by a hematoma or potentially seen directly on gray-scale US. However, it is often helpful to employ power/color Doppler as blood flow may be locally interrupted or at least abnormal appearing when a fracture is present.

## Data Availability

This are aspects evaluated qualitatively by the permer and there is no data to include.
